# Special Issue of the Manufacturing Engineering Society 2021 (SIMES-2021)

**DOI:** 10.3390/ma15144772

**Published:** 2022-07-07

**Authors:** Álvaro Rodríguez-Prieto, Francisco Javier Trujillo

**Affiliations:** 1 Department of Manufacturing Engineering, Industrial Engineering School, Universidad Nacional de Educación a Distancia (UNED), St/Juan del Rosal 12, 28040 Madrid, Spain; 2 Department of Civil, Materials and Manufacturing Engineering, EII, Universidad de Málaga, 29071 Málaga, Spain; trujillov@uma.es

After the complete success of the first [1], second [2,3], and third editions [4,5] of the Special Issues of the Manufacturing Engineering Society (SIMES), with a total of 118 contributions (94 in *Materials* and 24 in *Applied Sciences*) on emerging methods and technologies, the Special Issue of the Manufacturing Engineering Society 2021 (SIMES-2021) [6,7] was launched again as a joint Special Issue of the same journals—*Materials* and *Applied Sciences*. 

Once again, this Special Issue was promoted by the Manufacturing Engineering Society (MES) of Spain [8], with the aim of covering the wide range of research lines developed by the members and collaborators of the MES and other researchers within the field of manufacturing engineering. The Special Issue aims to explore the evolution of traditional manufacturing models toward the new requirements of the Manufacturing Industry 4.0, addressing how manufacturing professionals should face competitive challenges in the context of an ever-increasing use of digital information systems and communication technologies.

In this fourth edition, the joint Special Issue has gathered a total of 10 contributions (9 papers and 1 review) in the topics presented in Figure 1, where the percentage of contributions to each topic of the Special Issue from the Manufacturing Engineering Society 2021 (SIMES-2021) is also shown.

Regarding the specific contributions to the Special Issue in the *Materials* journal, six papers about cutting-edge advances in different fields of manufacturing engineering have been published after a rigorous review process. In particular, contributions have been published in “Advances and innovations in manufacturing processes”, “Robotics, mechatronics, and manufacturing automation”, and “Micro- and nanomanufacturing”. Figure 2 shows the main topics and their percentages in this journal.

Among them, the topic “Advances and innovations in manufacturing processes” received the highest number of contributions (representing 66.67%), followed by the topics “Robotics, mechatronics, and manufacturing automation” and “Micro- and nanomanufacturing” (each at 16.67%). 

Concretely, within the topic “Advances and innovations in manufacturing processes”, in the field of welding, García González et al. [9] presented a study of the consequences of keeping a fixed-position distance for the orbital TIG machine on the weld quality of the tube-to-tubesheet joints. In order to achieve their objective, they built two header boxes and carried out their metrological control in terms of parallelism deviation. Subsequently, two coupons or mockups were designed and welded simulating these measured deviations in the welding process of tube-to-tubesheet joints. All the welded joints of the mockups were made according to a standard procedure for the petrochemical industry, using two preheating temperatures for each case.

Another interesting work in this topic, in the field of simulation and modeling of manufacturing processes, is presented by Fernández et al. [10]. The objective of this work was to investigate the effect that the selection of the die material generates on the extrusion process of bimetallic cylindrical billets combining a magnesium alloy core (AZ31B) and a titanium alloy sleeve (Ti6Al4V). This work is of interest in aeronautical applications. A robust finite element model was developed to analyze the variation in the extrusion force, damage distribution, and wear using different die materials. The obtained results can potentially be used to improve the efficiency of this kind of extrusion process and the quality of the extruded part that, along with the use of lightweight materials, can contribute to sustainable production approaches. Torres-Escobar et al. [11] developed an experimental investigation to determine the compression behavior of hybrid steel tubes formed by two concentric steel tubes and four different fillers of non-metallic material, interposed between both tubes: polyurethane foam, polyurethane, epoxy, and a cement-based mortar. The tests showed that the incorporation of a resistant filler in the double tube allows it to improve its mechanical behavior by allowing a second load cycle. Furthermore, the strain energy absorbed during the two cycles led to the conclusion that the epoxy-filled tube absorbed more energy per unit of weight than the other resistant fillers. On the other hand, Blanco et al. [12] performed a rigorous review about the current trends in materials and processes in lightweight structural materials for aeronautical and automotive applications with a sustainable perspective.

Related to the topic “Robotics, mechatronics, and manufacturing automation”, the work by Calvo and Gil [13] introduced two new parametric models for collaborative robotics, formulated in order to evaluate the differential cost of assembly (economic dimension) and the differential income from taxes that supports short-term workforce displacement (social dimension) in cobot implementation.

Finally, the topic “Micro- and nanomanufacturing” received a contribution by Zamora et al. [14] within the field of metal additive microfabrication. This work represents the first analysis of the surface properties of this low-melting-point metal alloy and its suitability for use in applications associated with droplet deposition. Only seven months after the publication of the first work [12], all the papers present prominent activity in their “article metrics”; it is remarkable how some of the papers belonging to this Special Issue have more than 3.800 abstract and full-text views, at this time, which provides clear evidence of the interest in all of these topics among readers of *Materials* in general, and among scientists and professionals from the industry in particular.

## Figures and Tables

**Figure 1:**
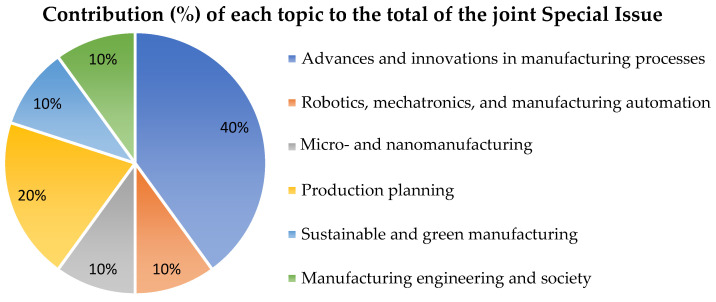
Contribution (%) of each topic to the total of the joint Special Issue.

**Figure 2:**
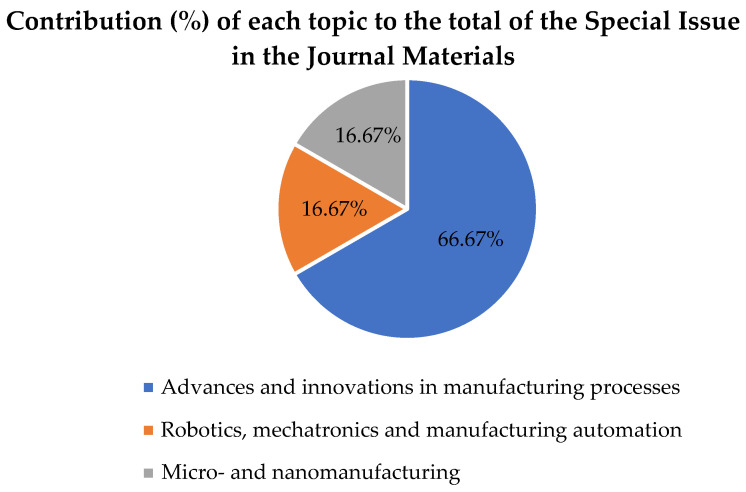
Contribution (%) of each topic to the Special Issue in *Materials*.
